# Editorial: Material Surfaces and Interfaces at the Nanoscale: From Theory to Application

**DOI:** 10.3389/fchem.2021.656661

**Published:** 2021-03-12

**Authors:** Dan Xia, Qiang Li, Shuai Zhang, Mingdong Dong

**Affiliations:** ^1^ School of Materials Science and Engineering, Hebei University of Technology, Tianjin, China; ^2^ School of Chemistry and Chemical Engineering, Shandong University, Jinan, China; ^3^ Department of Materials Science and Engineering, University of Washington, Seattle, WA, United States; ^4^ Interdisciplinary Nanoscience Center (iNANO), Aarhus University, Aarhus, Denmark

**Keywords:** material surfaces and interfaces, nano scales, Interdisciplinary research, advanced materials, nanotechnology

Material surfaces and interfaces at the nano scales have become an engaging subject of interdisciplinary research due to numerous promising applications in the last 2 decades. Highly sophisticated techniques and novel families of materials have emerged with explosive growth and convincing functions in catalysis (Jiang et al., 2021), energy (Janek and Zeier, 2016), environmental sciences (Kartal et al., 2010), biomedicals (Zhang et al., 2020; Xu et al., 2021), *etc.*. Developing theories behind observed materials performances is also vital to the sustainable success of this interdisciplinary field and the successful implementation of new materials and processes into the next generation of advanced materials.

In this special issue, we introduce the research focusing on the structure, properties, and technical applications at material surfaces and interfaces within nanoscale. The collection is dedicated to interdisciplinary research papers, integrating knowledge and practices of materials science, bio-related sciences, and chemistry into critical applications. Two research papers and three reviews are included in this special issue, which provides readers with selected cases of how the theory and techniques of nanoscale material surfaces and interfaces can contribute to material chemistry advances in various aspects.

The first research article, authored by Michal Otyepka’s group from Palacký University Olomouc, Czech Republic, focused on the nanostructure of the materials surface and interface. The synthesized graphene-iron carbide hybrid possesses a hierarchical structure with nanoscale pore size. This novel structure lead to the fascinating performance with a satisfying detection limit when it is used to detect dopamine within ascorbic acid. This indicates that the nanostructures on the materials surface and interfaces is vital to the outstanding performance of advanced materials.

The second research article, authored by Chengzhi He’s group from Beijing University of Chemical Technology, China, is concentrate on the technical application at materials surface and interfaces. The interaction force between a silica-binding peptide SB7 and glass surface is observed by the cutting-edge single-molecule force spectroscopy, and the underneath theory is revealed by the molecular dynamics simulation. This study shows the selection of appropriate techniques is key to reveal the mystery of materials surface and interface at nanoscale, thus the distinguish performance of the novel materials.

The three review articles emphasize the combination of materials science, bio-related sciences, and chemistry at surface and interfaces for biomedical applications. Chenxuan Wang’s group, from Chinese Academy of Medical Sciences and Peking Union Medical College, reviewed the devising of the protein building blocks for programmable hierarchical structures. Lei Liu and colleagues from Jiangsu University, China, focused on the newly photosensitizing materials and discussed photodynamic therapy techniques for the possible application in the treatment strategy of amyloid-related diseases. Ning Li and Ruodan Xu’s group from the China Academy of Chinese Medical Sciences, review the applications of functional biocompatible nanomaterials in improving curcumin bioavailability. All the review articles highlight the key role of material surface and interface in the design and synthesizing of novel materials, and further obtaining the desired performances.

Synthesizing and exquisite controlling materials with desired length scales will result in developing of entirely new classes of materials with tunable properties (Wang et al., 2020). These new arose materials are applied in various field, which lead to the explosion of new devices and technologies that could not been imagined only a few decades ago (Jiang et al., 2018; Yang et al., 2020). From this concise collection, we have witnessed the latest developments of material surfaces and interfaces at the nanoscale in diverse aspects of materials chemistry and science. We hope that the collection will attract more scientists from different research fields to join in so as to promote the frontier of material surfaces and interfaces research.

We appreciate all the authors, reviewers, and editorial assistants who contributed to implement this special issue. We hope that this collection can provide readers with a conspectus of the achievements and progress of material surface and interfaces at the nanoscale.
